# On the Influence of Electrical Fluctuations as a Cause of Disease

**Published:** 1857-05

**Authors:** S. Littell

**Affiliations:** Surgeon to Wills Hospital for the Diseases of the Eye and Limb, Philadelphia


ART. I.—*On the Influence of Electrical Fluctuations as a Cause of Disease.* By S. LITTELL, M. D., Surgeon to Wills Hospital for the Diseases of the Eye and Limb, Philadelphia.*

THE immediate occasion of the present Paper—which in much both of form and substance has already been made public—was information from the late editor of the Medical Examiner, that he had received from Mr. Craig, of Ayr, in Scotland, a letter alluding to an article published by me in that Journal, expressing his concurrence in the views which it contained, and referring to an accompanying pamphlet, in which his own opinions were more fully detailed. His communication furnished opportunity and pretext for bringing the whole matter before the Society; and I have condensed my several papers into one, thinking that my humble essay, thus supported, would be better received, and more thoroughly considered, than it was likely to be under other circumstances. What I have thought and written may not produce in other minds the conviction which has been wrought in mine; but an independent observer, regarding the subject from a different position, may supply facts and reasoning which I have omitted, and thus give to it greater interest and authority. I have not yet read the pamphlet alluded to, and am entirely unacquainted, therefore, with the ground it covers, or the argument which it contains; but the gentleman to whom it was addressed, will, before our adjournment, either read the whole, or such extracts from it, as may be necessary to place it fully before the Society.

In no department of medicine is there more crude and unfounded theory than in that which treats of the etiology of disease. Theories framed in the infancy of science, and transmitted unquestioned from one generation to another, are still blindly adopted and implicitly followed; though their inconsistency with facts of daily occurrence, can hardly have escaped the

* Read before the Medical Society of Philadelphia, Jan. 14, 1857.

observation of the intelligent and reflecting. The opinions generally prevalent on this subject are, indeed, hardly creditable to us as members of a learned profession, because they prove that we have not been guided in our reasoning by sound principles of philosophy. It is a maxim in science to assign no more causes than may be necessary to produce the effect; but we have disregarded this obvious restriction in a department of knowledge where it should have been more especially observed, and instead of taking a comprehensive survey of the action of morbific agents on the human system as a whole, have limited our attention too exclusively to the various pathological results, and needlessly invented a different cause for almost every aberration from the healthy state. Simplicity is found to be an attribute of the Almighty in all the operations of His hand; we are amazed at the number and diversity of the effects produced by the combinations of a few simple elements; and have reason to believe that as our knowledge increases, this characteristic will be still more apparent. Why should not the same be true also of the animal economy? It is a complex and intricate organism, composed of many different tissues, but all subjected to the control of a central power—the brain,—from any change in the action of which, innumerable deviations from a normal condition might, *a priori*, be anticipated. How much more philosophical then, to recognize a single principle capable of producing such change, than unnecessarily to multiply causes, and invoke the interposition of as many agencies as there are diseases in the nosology! We have imaginary miasms,—many of them supposed to be cotemporary in their existence,—for the several exanthemata, for influenza, for cholera, for dysentery, for each of many different kinds of fever, for hooping-cough, parotitis, &c. &c. In accounting for the phlegmasiæ, it is true, we are contented to veil our ignorance and flatter our vanity, under the convenient and comprehensive phrase of "taking cold;" an expression, however, to which we attach no definite ideas, and which, in its literal sense, the commonest observation shows to be incorrect.

An etiology so manifold cannot be true; and if the abnormal manifestations may, in very many cases, be more satisfactorily explained through the instrumentality of a single principle, it must be abandoned.

The analogy existing between the nervous force and electricity, first observed by Galvani, has been abundantly demonstrated by Dr. Philip, Majendie, and other physiologists, in their experiments on animals. Their actual identity, indeed, has been rendered not improbable; for nervous communication having been interrupted, the processes of digestion, respiration, and circulation are, for a time, performed as usual under the influence of electricity. It was, moreover, soon ascertained to have the power of exciting muscular contraction in bodies recently dead; while its remedial agency in certain complaints, especially those of a neuralgic character, has long been known; and with these facts to provoke and guide inquiry, it is surprising that the possible pathological consequences resulting from its deficiency in the atmosphere, or its rapid abstraction from the system, have not been more generally suspected and investigated. The arguments adduced by some physiologists to disprove its identity with the nervous force, and to establish instead, a state of correlation, are far from being conclusive; and even if this were admitted, it would not militate against the view I propose, which only requires such an affinity, or relation, that one shall be influenced by changes in the quantity of the other. Caloric, light, and electricity, are now regarded as probable modifications of the same element, and there is no reason why, under another modification, that element should not constitute the vis nervosa also. A strong presumption of their substantial sameness, is afforded by the existence of several species of fishes with electrical organs; the action of which is dependent upon their connection with nervous centres, varies in intensity with the extent of that connection and the health of the animal, is under the control of the will, and by a continual series of discharges is capable of exhausting the nervous energy to a degree sufficient, in some cases, to occasion death.

The electrical fluid is the grand agent in the production of many of the phenomena of nature. In her inorganic domain it is the probable cause of all chemical change, while in the vegetable kingdom it performs still more important functions; not only producing, in conjunction with its kindred agencies of light and heat, conditions favorable to the germination and growth of plants, but quickening them into life, and thus becoming the efficient cause of their development. Its intense and rapid passage—as in the lightning stroke—immediately kills the largest tree, and a very small shock sent through certain plants, will speedily cause their leaves to droop, and as certainly, though more slowly, extinguish their vitality. The approach of an electrified conductor to the mimosa pudica, or sensitive plant, produces no sensible effect, but if sparks be taken from it, the leaflets will shrink and close, as they do from mechanical contact. Its more steady and quiet operation is equally remarkable. An electrical circle has been formed by wires running under the beds of a garden, and the result has been greater vigor and rapidity of growth in the plants which they contained. It evidently performs the part of a general stimulant; which, when in moderate quantity, is salutary in its effect on vegetable life. May not the folding of the leaves of certain plants, as the mimosa, trifolium repens, &c., at the approach of evening, be owing to the abstraction of electricity which takes place at that time from the increased conducting power of the air?

A fluid so analogous to the nervous foice, so subtle, fluctuating, and so universally diffused, might, *a priori*, be supposed to exert a very manifest influence over the higher organization of the animal economy. Its presence in a certain degree, seems indeed to be necessary for the healthy performance of all the functions. The conducting power of the nerves has been shown by the physiological experiments alluded to, and the anatomical structure of the brain, with all the phenomena of nervous function and action, would naturally lead us to regard the whole nervous system as an apparatus, through the medium of which, electricity, modified and restrained by certain laws, is made subservient to the purposes of existence. In other words, as a vital electrical machine, by means of which that fluid is both separated and distributed in accordance with the wants of animal life. What we should thus suppose, is found to be true in fact; electricity when present in excess, exciting the functions and exalting vitality, while a contrary effect is produced by its subtraction or deficiency. In a state of health and mature existence, when all the functions are vigorously performed, and the power of resisting noxious agencies is greatest, the disturbing influence of such fluctuations is comparatively slight; but under other circumstances they become a frequent and potential cause of disease. We have all experienced the feeling of energy and elasticity which is imparted by what we call bracing weather, when the air is clear, dry, and cold; and more strongly still, the sensations of chilliness and discomfort, when the atmosphere, loaded with moisture, has acquired an active conducting power, and its injurious operation is further increased by the agency of cold; which, under such circumstances, has a depressing, instead of a stimulating effect. The nervous system of some susceptible individuals is thrown into commotion by an approaching storm; and I have a patient, for many years the victim of an annual catarrh, whose sufferings are always greatly aggravated by the occurrence of thunder at any time during the paroxysm. Rheumatic, neuralgic, and paralytic persons, and those who have recently suffered from sprained or fractured limbs, can predict with unerring certainty, an impending atmospherical change;* and the evening exacerbations which we observe in fevers and other complaints, are owing to the same cause; the system in its disturbed or debilitated condition being unable to bear, without suffering, electrical changes which would have little or no perceptible influence in a state of health. The pain of rheumatism, neuralgia, and the uncomfortable sensations accompanying catarrh,

* It is worthy of remark that these effects are chiefly produced in the elemental changes which precede a storm; when the snow or rain begins to fall, the electrical equilibrium is restored, and, if the vascular system has not become involved, neuralgic pains and uncomfortable sensations subside.

are in like manner, and for the same reason, all aggravated by the approach of evening.

It is these changes, moreover, consequent upon the withdrawal of the sun's rays, by which the dew is precipitated, and the conducting power of the air increased, which render exposure at this time so dangerous in certain districts of country, in the early autumnal months; and not as has been supposed, the greater prevalence on such occasions of miasmatic exhalations. At a later period of the evening, when the dew has actually fallen, the atmosphere being drier, is less penetrating, and exposure, consequently, less injurious. The early morning air, though charged with the ascending dew, is less deleterious, because, among other reasons, it acts upon a body in some measure invigorated and refreshed by sleep; but its influence, in debilitated states of the system more particularly, is nevertheless prejudicial; and hence the recommendation to avoid exposure on an empty stomach, the prescription of bitters, &c., which act by imparting temporary energy to the frame. The extreme sensibility to electrical fluctuations, of the affections which are purely nervous, is a subject of common observation. Every physician must have observed the greater frequency of asthmatic attacks before a change of weather. The epileptic paroxysm occurs most frequently in the night; and while this may, perhaps, be explained in part, by the temporary suspension of the will, and congestion of the brain in sleep, it is not irrational to attribute it in some degree, to the electrical changes which occur at that period; especially as we know that some persons subject to this malady, are only affected at the vernal and autumnal equinoxes, when these changes are greater than at other times. Rheumatism and neuralgia, hardly excepted, there is not a disease in the whole catalogue, the phenomena of which are in more obvious harmony with the electrical theory than those of epilepsy. The periodical accumulation of excitability, and its exhaustion by the paroxysm, forcibly recall the circumstances attending the charge of the Leyden Jar.

The familiar expression of "taking cold," which is supposed to account so satisfactorily for many of the ills to which flesh is heir, may be mentioned as another example of such influence. This is owing, not to variations of temperature, as generally believed; but to disturbances of the electrical equilibrium, of which these variations are the effects or accompaniments. It is not unusual for individuals, especially those of tender age, to retire in apparent health to rest, in a comfortable room and bed, and to awaken after some hours, with a sore throat, or a paroxysm of croup; and we have all known persons to be attacked with these and other complaints, said to arise from "cold," who have been closely confined for days or weeks to apartments, the air of which has been steadily maintained at an elevated temperature. They could only have been affected, therefore, by changes in the electrical constitution of the atmosphere without, and these would be felt with the instantaneousness of thought, however the individual might be situated and protected.

The effect on the gravid female of certain atmospherical conditions, has long been observed: "cold, rainy weather, and low, damp, miasmatic localities," says Professor Gilman, "have been recognized since the days of Hippocrates, as disturbing pregnancy and causing abortion. To the influence of the atmosphere is to be attributed the frequency of abortion, or other mishap in pregnancy by which some years are signalized." The probable explanation is, that the expenditure of the nervous energy in the reproductive process, renders the system more liable to be affected by electrical changes; which again are increased by the greater conducting power of the atmosphere in the places and seasons mentioned. Insane persons, on the other hand, have always been in a remarkable degree insensible to atmospherical vicissitudes, as well as free from epidemical influences; and this exemption is due to the habitual exaltation of cerebral action in their case.

These, and a host of similar facts may be adduced to prove that there is nothing improbable in the hypothesis, that under circumstances of predisposing, or concomitant influence, general in their operation, or affecting the individual only, pathological consequences of far greater gravity, variety, and extent, may be occasioned from the exhaustion of nervous energy by the subtraction of electricity through changes in the distribution of that fluid; and that the exhaustion thus induced, may be the proximate cause, not only of the exanthemata, and of most other forms of fever, congestive, or otherwise; but also of cholera, influenza, hooping-cough, erysipelas, dysentery, parotitis, the idiopathic phlegmasiæ, &c. &c. Among the circumstances alluded to, as affecting individuals, and thereby giving efficiency to electrical fluctuation, may be enumerated, fatigue, fasting, loss of sleep, the depressing passions, and whatever tends to debilitate the frame, and exhaust or diminish nervous power.

To my mind the conclusion is irresistible, that an element which thus pervades all nature, and plays a part so important in all her operations; which is so analogous to the nervous force that it may even be substituted for it in the performance of its appropriate functions; which, when in usual quantity, maintains the organism in healthy action, and stimulates or destroys according to the degree of its excess; must by its deficiency or subduction, especially in debilitated states of the system, and when aided by any cause tending of itself to depress cerebral action, exert a far more potential influence for evil.

The long continuance, in various degrees and combinations, of heat, cold, drought, and humidity, or the marked predominance of any one of these conditions, will create predispositions which determine the character of the prevailing diseases. A hot and dry summer, for instance, will be nosologically distinguished by affections of the alimentary canal. A higher grade and longer continuance of heat, producing a greater degree of exhaustion, and occasioning a strong tendency to inflammation of the stomach, liver, and other organs, is the predisposing cause of yellow fever. In the early autumnal months, the stimulus of light and caloric being lessened, while the system, exhausted by the previous heat, remains weak and impressible, and, therefore, easily affected by electrical changes,—which are promoted by various meteorological circumstances, as cold, humidity, &c.,—intermittent and remittent fevers, in which the pathological condition is rather congestive than otherwise, chiefly abound. The cerebral functions are impaired, innervation is lessened, vascular congestion takes place, and reaction following, the usual febrile phenomena are developed, which assume an intermittent, remitttent, continued, or typhoid form, according to the intensity of the cause, or the degree of the pre-existing debility. A peculiarly raw and searching atmosphere precedes and accompanies influenza, a disease which is characterized by excessive nervous disturbance and debility; while an open, wet, and variable state of the weather, such as we frequently see in November, is favorable to the production of the exanthemata, typhoid fever, erysipelas, &c. The reaction occasioned by a higher degree of cold, the air being dry, and having, therefore, little or no conducting power, is salutary and invigorating.

In our reasoning on this subject, the effect of the diminished light and heat occasioned by the sun's southern declination, must not be overlooked. If this diminution be sufficient to suspend vegetable life, and convert the beauty of earth into the gloom and severity of winter, it is surely no unreasonable supposition that it must also exert an influence injurious to animal existence; which would be more sensibly felt, but for the reaction it calls forth, and the power of accommodation which the organism possesses. The illuminating and heating rays of the solar beam are those which exercise the greatest apparent power over the human frame. They act in their intensity as powerful stimuli, exciting the circulation, and exhausting the vital force in a remarkable degree; and according to a well-known law, their withdrawal, or considerable diminution, must necessarily be followed by a depression corresponding to the previous exaltation.*

The view which I have taken of this subject is not wholly speculative. The diminution of magnetic power during the prevalence of cholera, has

* From the sevenfold nature of the sunbeam, we should reasonably infer the possession by each ray, of a different virtue, or property. That of four of them has been already ascertained,—the lighting, the heating, the chemical, and the phosphorogenic property.

been ascertained by direct experiment. Mr. Mather, of South Shields, England, states that in 1849, when cholera of a very fatal character was epidemic in his neighborhood, he found, as the result of numerous observations carefully made, that a magnet which ordinarily carried two pounds and ten ounces, would, when the atmospherical indications were nearly at their worst,—the air being saturated with moisture—sustain only one pound and ten ounces; the degree of its attraction varying with, and being in inverse proportion to, the virulence of the disease.

The same year the number of deaths in Paris, from this pestilence, rapidly increased until the eighth of June, when they amounted to six hundred and twenty-three. On the evening of that day there occurred a thunder-storm of unusual severity, and the cholera immediately began to decrease; by the eighteenth of the month there was a daily report of one hundred only; and at its close, the mortality had fallen to thirty. Similar observations have been recorded by others; and from the consideration of all the circumstances attending this disease, with its preference for the great water-courses of a country, &c., its dependence upon a state of deficient electricity may be regarded as pretty certainly established. Whether operations going on in the interior of the earth, do not influence the electrical condition of its surface, is a subject which may demand investigation.* The prevalence of cholera during the past year in Sicily, Madeira, and Central America,—all of them volcanic countries,—would seem to give some plausibility to the conjecture. The air of the Pontine marshes, near Rome, so fatal to those within its influence, is deficient in electricity, and possibly from the same cause. But it may be remarked, in passing, that the air of marshes, and still water generally, is more prejudicial than that of rivers or running streams; partly for the reason, that the air, saturated with moisture, undisturbed by atmospherical currents, and possessing, therefore, more active conducting power, lingers upon them in unbroken mass, long impervious to the rays of the sun; and partly, because, in the latter case, the motion of the particles among themselves, and their friction against the hills, trees, &c., when driven by the winds, is itself a principal cause of atmospherical electricity. It is probably in both of these ways, that the agitation of the air, by the frequent passage of a steamboat—itself a most active hydro-electric machine,—increases the salubrity of places in its vicinity, or restores it when lost; as, for instance, in the case of the Schuylkill above the dam at Fairmount. The meteorological constitution in which influenza appears, would incline us to predict with confidence the origin of that complaint in defective electricity; and most of the diseases to which I have before alluded, are notoriously most frequent in sea-

* The proximity of such operations to the surface of the earth, and the nature of its crust influencing its conducting power, would, of course, render some places more liable to be affected than others.

sons of the year when electrical currents and changes are greatest, and their injurious operation aggravated by moisture and other auxiliary influences. There are many facts, moreover, irreconcilable with the commonly received notion of the malarious production of fever; and without absolutely denying the deleterious action on the animal economy, of exhalations from decaying vegetable matter, I am fully convinced that these are not the general cause of the disease, and that a part far too prominent and exclusive, has been attributed to their agency. The theory afforded a plausible solution of many things hard to be understood, and being supported by a multitude of seeming facts, has been too hastily received and adopted by the profession. The complaints supposed to be thus engendered, prevail at a season when electrical vicissitudes are greatest, and the body, debilitated and otherwise disordered by the protracted heat of summer, is most sensible to their impression; they are often observed where there is no reason to suspect the operation of malaria; are notoriously reproduced by other causes after they have once occurred; and are promptly cured by means which eliminate no poison, but merely restore the lost tone of the system,—frequently, indeed, by mental impressions alone.

Were it true that intermittent and remittent fevers owe their origin to paludal exhalations, or to malaria, however generated, we should naturally expect to find them most prevalent when vegetable decomposition is greatest; but a moment's reflection will show that the reverse of this is true. In the Middle and Western States, and perhaps throughout our country, September is the sickliest of the autumnal months; and yet vegetable life still flourishes, often in almost undiminished vigor; the foliage preserves its verdure and freshness, and nature exhibits few symptoms of her approaching decay. The days, moreover, have become considerably shorter, the weather cooler, and it is evident, therefore, not only that *materiel* for decomposition is not supplied in greater abundance, but that the causes which concur in that process, are really less active than they were in the preceding months. Those seasons, moreover, which are characterized by an unusually late fall,—vegetation being fostered by timely rains, and long unchecked by frost,—are precisely those in which autumnal fevers prevail more extensively, though, owing to the system being less exhausted by heat, of a milder type, than under other circumstances. The year 1855 may be adduced in illustration. Summer and autumn were both marked by cool, wet, and variable weather; the country, perhaps, never preserved its freshness to so late a period; and yet, intermittent and remittent fevers were more than ordinarily frequent, not only in localities where they are usually met with, but also on elevated grounds, celebrated for their salubrity, and even in the very heart of the city. Circumstances like these, cannot be accounted for on the theory of malarious exhalation, but receive an easy solution from the agency of humidity in increasing the conducting power of the atmosphere, and thus giving greater effect to electrical changes.

That intermittent and remittent fevers prevail epidemically in the fall, and occur only in sporadic cases during the spring, is owing to the circumstance, that in the former case, the system, unduly stimulated by the heat of summer, and left in a state of exhaustion and debility by its withdrawal, is less able to resist the electrical fluctuations which are the efficient cause of their production. Seasons in which the warm weather has been unusually protracted, and the winter uncommonly mild and open, are precisely those in which the most desolating epidemics have occurred; for the reason that the system, uninvigorated by cold, falls a ready prey to the action of the cause which I have suggested. In some climates, as in that of Guayaquil, this is the invariable state of things. The winter is the period of almost incessant rain for six months' duration, and the mortality is, consequently, very great.

The human frame possesses a great power of accommodation to external agencies, especially when these are uniform and constant in their action. The air of the sea, and generally of places in its vicinity, though saturated with moisture, is healthy for this reason; for this moisture being general and invariable, tends to maintain the electrical equilibrium, though perhaps at a lower range; while the system being less enfeebled and disordered by heat, the atmosphere purer and denser, and the nights cooler and more refreshing, such fluctuations as do occur are less sensibly felt. Hence also it is, that in a very rainy season, localities which have been the immemorial haunts of fever, become comparatively healthy; while upland districts rarely visited by it, suffer in their turn.

A striking instance of a country in which every circumstance of climate, soil, and atmosphere, might be supposed to unite in the production on a grand scale of paludal exhalation, is mentioned by Dr. Hooker in his Himalayan Journals. The delta of the Soormah extends for a distance of eighty miles along the old bed of the Burrampooter, a river five miles broad; and forms an immense still and narrow sheet of deep clear water, called the Jheels. The area drained by the Soormah is scarcely raised above the level of the sea, and contains about ten thousand square miles. In the dry season the Jheels are marshy, but during the rains, which are excessive on the neighboring mountains, they are entirely overflowed; the water rising to within a few inches of the huts which are built along the borders of the rivers that traverse it. The soil, sandy along the Burrampooter, is more muddy and clayey in the centre of the Jheels, with immense accumulations of vegetable matter in the marshes, consisting chiefly of decomposed grass-roots and leaves. "The climate of Chattuc," says the

Doctor, speaking of one of the villages, "is excessively damp and hot throughout the year, but though sunk amid interminable swamps, the place is perfectly healthy. Such indeed, is the character of the climate throughout the Jheels, where fever and ague are rare; and though no situations can appear more malarious than Silpat and Cachar, they are in fact, eminently salubrious. These facts," he continues, "admit of no explanation in the present state of our knowledge of endemic diseases. Much may be attributed to the great amount and purity of the water, the equability of the climate, the absence of forests, and of sudden changes from wet to dry; but such facts afford no satisfactory explanation." Undoubtedly they do not, on the supposition that malaria is necessarily concerned in the production of such complaints; but discard that hypothesis, and they receive obvious elucidation on the theory of their electrical origin. Humidity alone, when universal and constant, tends, as I have said, to preserve the electrical equilibrium; and the great extent of their surface gives to the Jheels the character of an inland sea; the steady warmth of the weather sustains the vital actions; while the circumstances mentioned by Dr. Hooker, must render electrical vicissitudes slight and infrequent; and hence their exemption from the so-called miasmatic diseases.

There is no circumstance indeed, connected with our autumnal fevers; their endemic, and occasional epidemic prevalence, the influence of moisture, the comparative exemption of large cities, the agency of winds, &c., which cannot be more scientifically explained on the electrical, than on the miasmatic hypothesis; and the former has the additional advantage of substituting an adequate, existing, and recognizable cause, for one which rests on no certain foundation, eludes all chemical scrutiny, and is to a very great extent, if not altogether, imaginary.

The objection to this view, that disturbances of the electrical equilibrium are of continual occurrence without being followed by such effects as are here attributed to them, is more specious than sound. It fails in not appreciating the consequences of the prolonged heat of summer, which, exhausting the nervous energy, leaves the system, in the early autumnal months, weak, susceptible, and predisposed to disease; and it is moreover, not altogether true in fact, for intermittent and remittent fevers are observed, though, of course, much less frequently, at other seasons of the year. As winter approaches, the invigorating influence of cold is felt in the increase of nervous energy; oxygen is breathed in greater quantity with a denser atmosphere; reaction follows the previous depression; and the predisposition changes to other forms of morbid action. It is to this circumstance, and not to the destruction by frost of malarious exhalation, that the cessation of our autumnal fevers should be ascribed.

I do not deny that the foul emanations arising from the decomposition of animal and vegetable matter, which vitiate the atmosphere of our large cities, are a concurrent cause of disease. They are often present in a degree sensibly offensive, and cannot be otherwise than extremely prejudicial to health. Such vitiation, and the impression of excessive and long-continued heat—the latter being absolutely necessary to its production,—are the great predisposing causes of yellow fever; and only require an atmosphere negatively electrical to give full effect to their injurious agency. The increased conducting power of the air when loaded with moisture, and its greater contamination from the embouchure of sewers, &c., account for the first appearance of the disease in the immediate vicinity of a river, or of the sea, upon which, places subject to it are situated. Yellow fever, as I have elsewhere observed, is an inflammatory affection, which expends its force generally and principally on the stomach and collatitious viscera, but may be complicated also with inflammation of other parts predisposed to increased action. It runs its course with great rapidity; and derives its fatality both from its involving vital organs, and from its being engrafted upon an exhausted state of the system.

The crews of vessels who have continued in good health while at sea, are often attacked by yellow fever, on arriving at ports where the circumstances which give rise to that disease, exist in high degree—though it may not previously have appeared among the inhabitants who have been in some measure accustomed to their influence,—and are unjustly charged with having brought it from the places whence they sailed. After a voyage, in which, from the constant impression of a moist atmosphere, the vital forces are rather depressed below, than elevated above their par condition, they not only exchange a healthy air for one rarified and impure, but their duties generally become more laborious and exhausting; and the system thus predisposed, with its energies perhaps further impaired by excesses of various kinds, is more readily affected by the electrical fluctuations which are engendered by the action of the sun upon the land. The earth, as we all know, receives and radiates far more heat than the water, and evaporation, with all the circumstances connected with electrical fluctuation and change, is more active along the line of separation.

That such diversity of effect should be produced by the same morbific agent, constitutes, as has been seen, no valid objection to the hypothesis which I advocate. Man, in his ignorance, is fond of multiplying causes, but science is daily demonstrating the simplicity of truth. In the present instance there is no necessity for a multifarious agency. The human frame is so constituted, that whatever impairs the energy of its controlling organ—the brain,—diminishes, of course, the supply of nervous power to all parts of the body; and this defective innervation may give rise to aberrations as various as there are tissues and organs to be acted upon. Its most common result is dilatation and congestion of the capillaries, with all the phenomena of inflammation. Thus, in the dermoid tissue, we may have derangement of the capillary circulation, or of the exhalants, or of its secretory apparatus; constituting respectively, scarlet fever, measles, and small-pox; or we may have cholera from the same cause directed to the mucous membrane of the stomach and bowels; or any one of the phlegmasiæ, according as the development of latent imperfection, or accidental causes may determine. To those, therefore, who consider the complicated organism of the human system, it will not appear strange that results apparently so different, and yet in reality essentially the same, should be produced through the instrumentality of a single principle, directed in its morbid manifestations by predispositions arising from a variety of circumstances, existing in countless combinations, and involving whole communities, or affecting individuals only.

The manner in which morbid action is produced by the abstraction of electricity from the system is sufficiently indicated by what has been already said; but it may perhaps be made still clearer by one or two additional examples; and scarlet fever, as being in its uncomplicated form, a familiar instance of a general inflammatory affection, will furnish an apposite illustration. This disease, though occasionally observed in every season of the year, prevails most extensively in autumn and spring,—periods during which electrical fluctuations are greatest, and their influence is promoted by the various meteorological circumstances so often mentioned before; and, as might be expected, is chiefly confined to children, whose power of resisting hurtful impressions, is less than that of persons of mature age and vigor. The action of the brain and nervous system being depressed, or otherwise disturbed by the rapid abstraction of sensorial energy, and their control over the capillaries lessened, these consequently become dilated; the circulation through them is retarded, and a state of things is induced, closely bordering on inflammation. This, though general in all the tissues, is more particularly observed in the mucous membrane of the digestive system, and in the skin; as evinced in the former by the redness of the throat, and the projecting papillæ of the tongue,—sometimes also by the occurrence of nausea or diarrhœa,—and in the latter by the scarlet efflorescence, and other symptoms of increased action,—the predominance of which, in this tissue, commonly indicate a tractable form of the complaint. Meanwhile, the brain reacting against the morbific agency, separates and transmits the nervous energy as before; but there is now a demand for a greater supply, in order to restore the impaired tonicity of the capillaries;* this restoration is accomplished through the exaltation of

* It is this demand requiring all the ability of the system to sustain, which renders depletion, and other measures producing still further exhaustion, so dangerous in the treatment of this disease.

its functions occasioned by the febrile movement,—an action of salutary tendency when it does not transcend the required limits,—and after a commotion of greater or less severity, occupying necessarily a nearly definite period, the system reverts to a state of convalescence. Such is the order of things in scarlatina simplex. In the anginose variety, the pathological alterations proceed one step further. The circulation through a portion of the capillaries is not only retarded, but absolutely arrested; congestion follows, and inflammation is set up in the fauces, where, from the laxity of the parts, and the exposed condition of the vessels, we should naturally expect to find it. In still more aggravated grades of the malady, whether owing to the intensity of the cause, feebleness of constitution, or some other circumstance affecting the individual, the powers of life are prostrated, in many instances beyond the capability of reaction; the brain being deprived of its nervous energy, delirium, or coma ensues; and after a struggle of varying duration, death generally closes the scene—often supervening in a very few hours.

The year which has just passed, presented meteorological conditions favorable to the production of this disease; and it has consequently prevailed very extensively. The winter was excessively severe, and the cold was protracted throughout the spring. The early part of the summer was very warm, with the coolest August that had been known for a long period of time. The autumn was open and variable; and the mortality towards its close amounted to more than forty cases a week. A few consecutive days of clear, cold, and dry weather, occurred in the early part of December, and were followed by a marked diminution of the disease,—the number of deaths for the week following being only thirty. It subsequently became chilly, wet, and variable, and the mortality again increased beyond any previous example. The whole mortality for the year, from this cause alone, was nearly or quite one thousand. In New York, it amounted to more than twelve hundred.

The origin of measles may be explained in like manner. The difference being that in scarlet fever, the morbific influence exerts its force chiefly on the capillary circulation; whereas in measles, while it implicates the pulmonary, rather than the gastro-mucous tissue, it receives from some predisposing cause,—perhaps a somewhat greater degree of cold, or the same degree acting upon a system less debilitated, as in the spring—a determination to a different part of the skin; probably its exhalant vessels. In small-pox another, and more secretory portion of this composite structure is affected, and hence its contagious character.

The application of the same mode of reasoning to the phlegmasiæ is sufficiently obvious. An individual, from exposure in a raw and damp state of the weather,—the physical powers being perhaps depressed by fasting, fatigue, or some other predisposing cause, becomes unwell, and is said, in common parlance, to have "taken cold." More correctly speaking, the sensorial energy has been abstracted from the system more rapidly than it could be generated without disturbance of the cerebral functions; the effect is felt in the diminished innervation of some organ, liable from congenital or acquired predisposition, to fall into diseased action; and as a consequence, inflammation takes place either in its parenchyma, or investing membrane, as circumstances may determine.

The exanthemata prevail chiefly in early life, when the nervous system is not only predominant and impressible, but there is, moreover, from their greater functional activity, a natural tendency to affections of the dermoid and mucous tissues; the predisposition inclining, in after years, rather to affections of the contents of the great cavities. The exemption from a second attack, though by no means so general as is commonly supposed, increases with the more confirmed and vigorous action of the brain; and these diseases, therefore, unless there be a strong constitutional tendency or the predisposing cause exists in a high degree, rarely affect adults.

When the meteorological conditions are favorable to the production of small-pox, even vaccination will confer no immunity. That it appears to afford protection under any circumstances, is owing partly to the more confirmed vitality and different morbid predispositions of later life, and partly, also, to the fact that the conditions alluded to, combined with other depressing causes, as insufficiency of food and clothing, imperfect ventilation, neglect of cleanliness, &c., which so greatly facilitate their action, are rarely seen in this country.

For the professional expression of this opinion, Mr. President, notwithstanding my avowed practical conformity, I have been publicly arraigned by an intelligent and respectable member of this Society, as "promulgating new and doubtful doctrines, calculated to cause misgivings as to the virtue of a 'widely-adopted and long-cherished' blessing," in which he has the temerity to say that medical confidence is still undiminished; though it is known to the veriest tyro, that its advocates, driven from the ground of perfect immunity, have been obliged to resort to a modified protection,—creating a varioloid disease to explain a degree of mildness which is owing to improved modes of living and more rational treatment,—and are, moreover, divided among themselves as to the necessity of the repeated, and even septennial employment of their supposed prophylactic!

I am far from supposing that electrical fluctuation is the sole cause of morbid action. Disease once induced, has, in some instances, the power of self-propagation, and often originates, moreover, from other causes, operating as well within as without the individual; but when it prevails epidemically, or in sporadic cases of complaints sometimes epidemic, when there has been no exposure to contagion, and on all occasions when it is said to arise from "cold," its etiology I believe to be as I have described. Whatever, indeed, impairs the nervous energy may operate in the same manner, and produce, under similar predisposing influences, the same results. I have seen cases of scarlet fever and variola arising, as I supposed, from fatigue alone; and I observe that Evelyn states in his Diary, that one of the Princes of the Royal Family was attacked by small-pox of a confluent and fatal character, after excessive dancing.

When the predisposition is wanting, even contagion will be sometimes nearly, or altogether inoperative, and people are often exposed to it with perfect immunity. Variola itself, under such circumstances, will not always spread beyond the individual affected; and may even give rise to disease of a different kind. I have seen cases of fatal congestive fever without the characteristic eruption, manifestly caused by attendance on the confluent variety of that disease.

The subject opens a wide field for observation and reflection, and will require, on very many topics, an entire reconstruction of medical sentiment. Several complaints now attributed to contagion, will be found to spring from a cause which no isolation could evade; demonstrating thus the inutility and folly, as well as the mischief of quarantine regulations. Not only malaria, but the whole tribe of atmospherical miasms, with vaccination, and several other widely-adopted and long-cherished opinions, are destined to fall before the more rational theory which it inculcates; while it cannot fail to exercise also, a beneficial effect upon practice, in making the preservation and restoration of nervous energy a prominent object of regard both in health and disease.

It is not to be supposed that an hypothesis which allows of no divided empire, but seeks to exalt itself upon the ruins of what composes so large a part of medical literature, supplies so much of medical phraseology, and has exercised an influence so controlling over medical opinion, will be received without strong opposition. A host of prejudices will start up in arms against it, and may deprive it in many minds of the consideration which it merits. These should not be permitted to interpose obstacles to the pursuit of truth. I ask for it only a candid and thorough investigation. If it be true, as I firmly believe, it will ultimately triumph; and if otherwise, it should be refuted and rejected. It has been held by me with increasing conviction for more than twenty years; and from the clear insight which it gives into much that without it would be dark and contradictory, I should be happy to impart the confidence which I feel, in equal degree to others.

The doctrine is fruitful in its practical applications, and involves whatever may protect the system against the fluctuations of this potent and all-pervading principle. It supplies us with an intelligible reason why, in the selection of a residence, we should avoid localities the air of which is habitually charged with moisture, and its conducting power thereby increased, whether from the nature of the soil, or the vicinity of water. It teaches us, for a similar reason, to avoid exposure, in feeble states of health, to the early morning and evening air of the country, when intermittent and remittent fevers are rife; and when such exposure is unavoidable, points out the propriety of sustaining the vital powers by a previous meal, and the exhibition of some tonic or stimulant. It explains how it happens that in certain seasons, and during the prevalence of certain winds, situations ordinarily salubrious become unhealthy. It instructs us, moreover, during the existence of any epidemic, to abstain from everything which may depress or exhaust the nervous energy, and to maintain the action of the brain in its accustomed, or even in increased vigor, as well by the stimulus of hope and confidence, as by the use of means which exert their influence more especially upon that organ; affording thus the probable rationale of belladonna and other prophylactics. Whatever, indeed, sustains and exalts the nervous power, must necessarily tend to avert disease; and in the autumnal season more particularly, when the system, before its reaction under the influence of cold, is left in a condition not unlike that of an inebriate from whom his accustomed stimulus has been withdrawn, the general prescription of some appropriate tonic might be expected to prove especially useful. In the late expedition up the Niger, a river so often fatal to previous explorers, the health of the crew was preserved in a remarkable manner by the exhibition of quinine, morning and evening, with other hygienic precautions; and the same course might be advantageously adopted by vessels arriving at our ports during the prevalence of yellow fever. It guides us, furthermore, to a right practice in many affections now somewhat empirically treated; warning us to abstain from depletory, and other disturbing measures, in scarlet, typhoid, and other fevers, where the nervous energy is already impaired through the debilitating nature of the cause, and the subsequent reaction is merely an effort of the vis medicatrix to restore the lost balance of the system. A want of due innervation being a primary deviation in the train of morbid action, it holds out a reasonable hope of subverting certain complaints in their incipient stage, by the administration of tonics, as quinine, in large doses, before the vascular system has become implicated; and forbids a resort to the lancet, at least until such implication has taken place, and reaction, permanently secured, threatens, by its excess, to endanger some important organ. It abates, in many instances, as in scarlatina and rubeola, the dread of contagion; and relieves us from the supposed necessity of purifying the blood by the elimination of an imaginary materies morbi. It suggests the necessity of adequate clothing of appropriate quality,—that is, of non-conducting materials, as woollen or silk,—and other precautionary measures, in the management of children; and finally, admonishes us of the importance, at all times, of preserving the vital forces in their best possible condition, and thereby of affording to the vis medicatrix full opportunity of accomplishing its recuperative tendencies.

It might perhaps be supposed, on first impression, that disorders originating in a temporary abstraction of electricity, ought to be cured by the artificial supply of that fluid; and this supposition would not be unreasonable if our bodies, instead of being living systems, were inanimate machines. In the actual constitution of things, however, other morbid actions speedily follow the temporary relaxation of cerebral or nervous control; various complications ensue; and effects are produced which can only be obviated or repaired in accordance with the laws which govern the animal economy alike in health and disease. I have not found electricity of much value in the treatment of disease, until the system had been brought, by other measures, nearly or quite to its par condition, and the suspended function only required an appropriate stimulus to call it into activity.

In what has been said, the substantial identity of the vis nervosa and the electrical fluid has been assumed; but as before observed, this is not necessary for the truth of the theory which I have advanced. It is sufficient that there should be such a reciprocal relation between them that fluctuations in the one, will produce a corresponding change in the other—the morbid alterations being accounted for with equal clearness on either supposition,—and thus much, I presume, will be conceded by all physiologists.

The theme is a prolific one, and the ideas thus imperfectly expressed, far from exhausting the subject, must be regarded as little more than suggestive. They are not wholly original; for the electrical origin of several diseases has by some physicians long been suspected, and as respects one of them—Asiatic cholera—well-nigh established; but I am not aware that the hypothesis has ever been so fully developed, or received so extended an application as I have given to it.

To my mind it harmonizes and elucidates many discordant and otherwise inexplicable phenomena; inculcates a rational and conservative practice; and while by its adoption we substitute a simple, intelligible, and effective etiology, for one complex, contradictory, and inadequate, we get rid of much of the fanciful theory and unfounded reasoning, which have so long bewildered and disgraced our profession.

It is certainly more philosophical, and more in accordance with the operations of the divine Author of nature, who produces effects the most diverse and wonderful, by the agency of a few simple elements, to ascribe the causation of the diseases we have been considering, to a single instrumentality, capable, from its potent, fluctuating, and all-pervading nature, of accomplishing, through the governing organ of the animal economy, all that is thus attributed to it; than, like the heathen of the olden time, to create a deity for every effect, and people the invisible realms of medicine with as many miasms as there are maladies incident to the human frame. The general predisposition, individual tendency, the negative electrical condition of apartments vitiated by respiration, and other depressing influences, will satisfactorily account for much that in many diseases has been attributed to contagion.

The fiction, that there are floating in the atmosphere, miasms which enter into the circulation by respiration or otherwise, acting as a poison to the blood, and severally producing scarlet fever, measles, small-pox, &c.,—for these diseases often prevail in the same neighborhood, or household, and, two of them at least, sometimes in the same person,—might be very well for the age in which it was invented; but in the light of modern science, is, in my judgment, as absurd, as I believe it to be unfounded.

It is gratifying to perceive that the medical mind, so long held in the leading-strings of authority, is beginning to break the flimsy fetters which have bound it, and to assert its prerogative of independent thought. I have conversed, of late years more especially, with intelligent physicians from various parts of our country, and have found in many of them a strong feeling of dissatisfaction with the opinions prevalent on this subject, though they knew not what better to substitute in their stead. Contradicted by facts of familiar observation and occurrence, they are evidently losing their hold on the inquisitive and reflecting; and must, ere long, give place to views more in accordance with the dictates of a sounder philosophy.

I impute no fault, and cast no discredit on those who have gone before, and from whom they have been derived to us. They lived before the era of modern discovery, when electricity as a science did not exist; when its action upon the animal system, and the marvels of the telegraph were unknown; and the theories which they invented to reconcile and explain the phenomena before them, were not behind the philosophy of their age; indeed, they may be said to have been far in advance of it, for their authority has remained unchallenged almost until now. The demerit is all our own, in that, enamoured of their antiquated lore, we have stereotyped their errors, and cling to them as pertinaciously, as though they were rigid deductions drawn amidst the brighter revelations in physical knowledge, which it is our privilege to enjoy.

Surely the discovery of electricity, its capability of being temporarily substituted for nervous power in the process of digestion, &c., its action in producing muscular contraction in bodies recently dead, and its instantaneous transmission along the telegraph wires, manifesting thus a striking analogy to the nervous force or energy, might have been expected to awaken inquiry, and exert a modifying influence on opinions formed long anterior to that period. The nerves, being themselves tracts of medullary matter, do not act as conductors merely, but separate or secrete the energy which they transmit, and probably govern the capillary system in some measure independently of the brain.

Electrical fluctuation, as already intimated, has been conjecturally assigned, with more or less confidence, as the cause of several complaints; but few attempts have been made to show by argument the grounds upon which such conjecture reposed. Dr. Wood, in his Practice of Medicine, states that Sir James Murray, so long ago as 1844, maintained, in an article published in the Dublin press of that year, that the true malarious agents are electro-galvanic currents and accumulations, which produce disease by disturbing the electrical equilibrium of the body; an opinion identical with that, which, with wider extension, I have advocated in the present essay; though by what reasoning it is supported by him, I am entirely ignorant, for I have neither seen his paper, nor any other on the subject; and what I have written, therefore, is the exclusive result of my own observation and reflection. I have even abstained from reading the monograph of Mr. Craig, in order that the matter might be brought before this learned body, as the elaborated conclusion of two isolated and independent minds, employed unknown to, and uninfluenced by, each other, in the solution of the same problem.

I have not aimed at demonstration; but I hope, Sir, that I have succeeded in creating such a presumption of the truth of the theory which I have advocated, as gives it a fair claim to attention, and I commend it accordingly to the consideration of the Profession.

